# Supplementary figures and images for: RNA polymerase II promotes the organization of chromatin following DNA replication
Source: EMBO Rep. 2024 Feb 12;25(3):1387–414. doi: 10.1038/s44319-024-00085-x (PMC10933433; doi:10.1038/s44319-024-00085-x)

Figure S1E

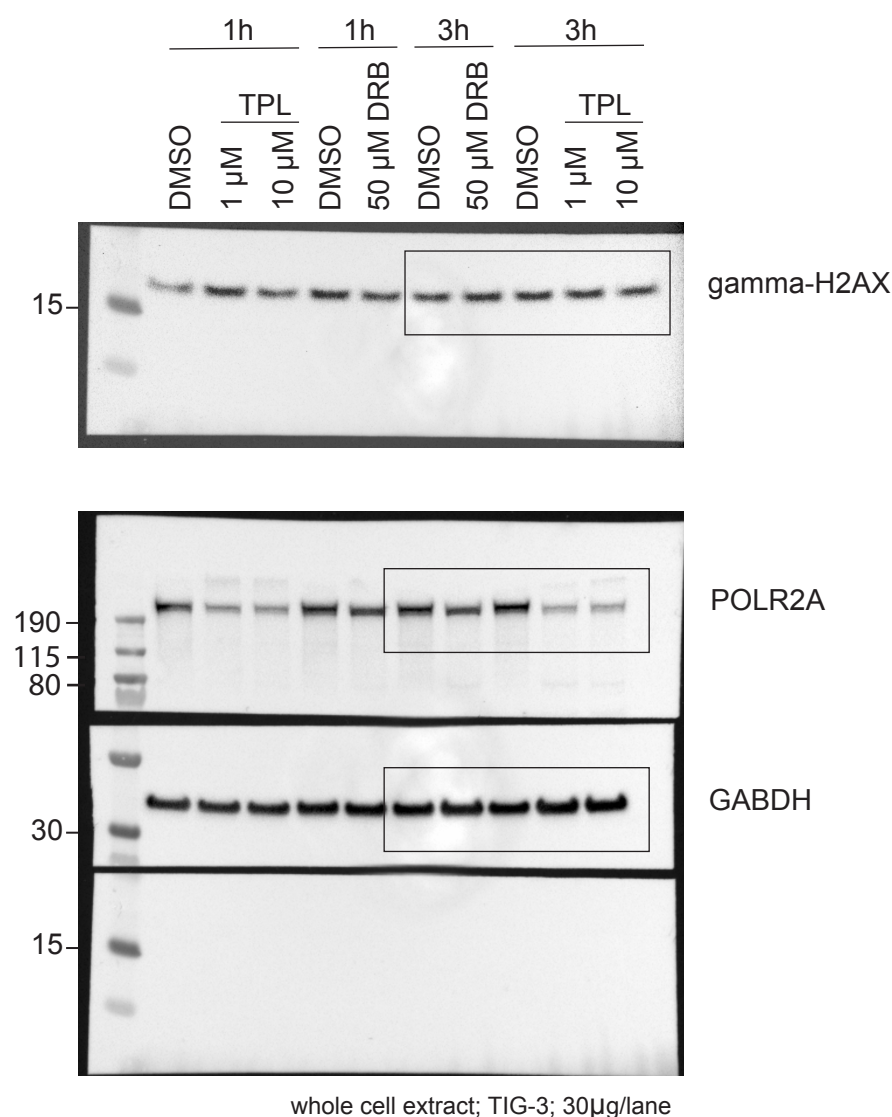

Supplement: Supplementary file 12 — EV Figures Source Data [file 44319_2024_85_MOESM12_ESM.zip › Figure EV1/E/README_FigureS1E.pdf]

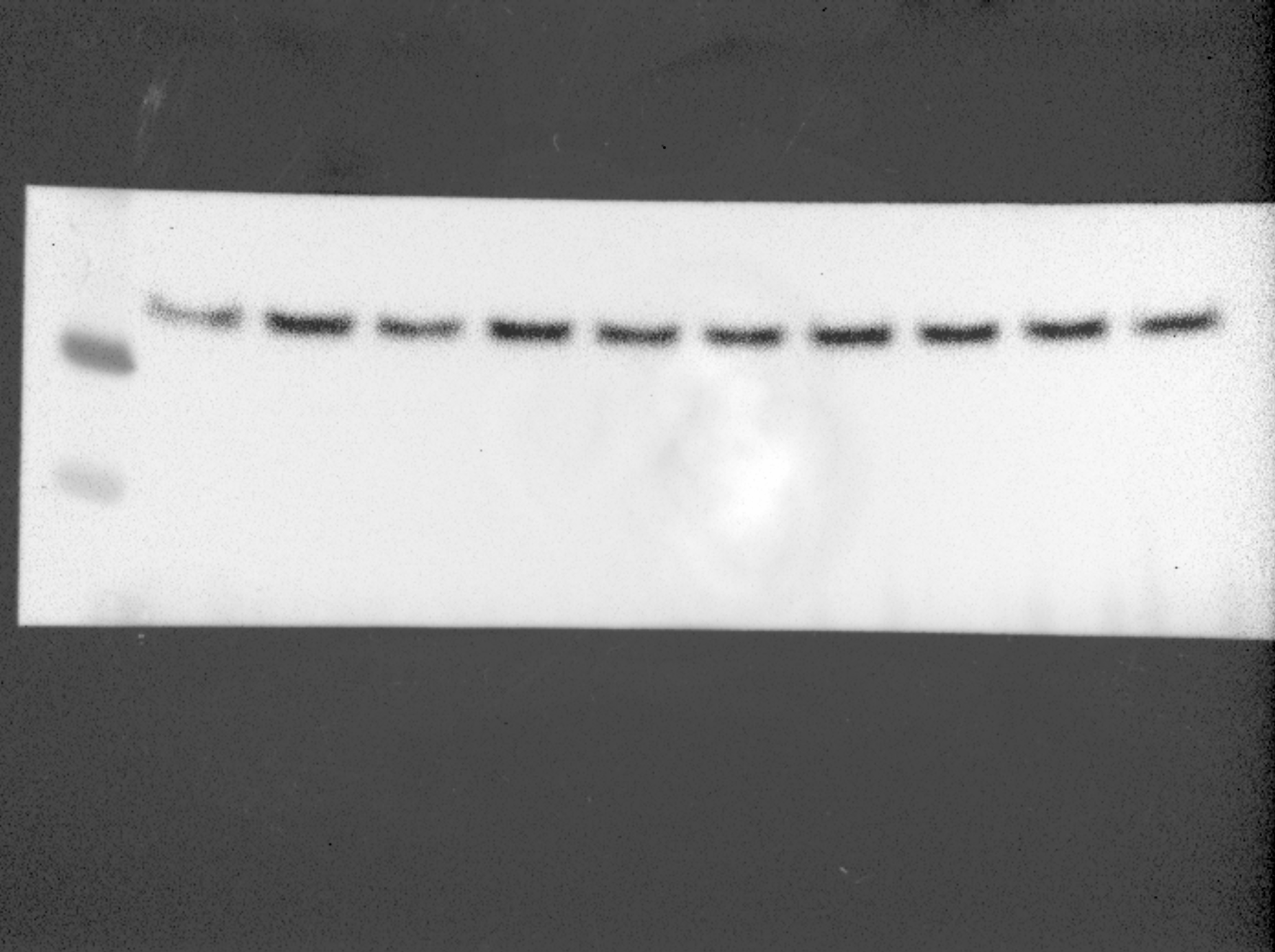

Supplement: Supplementary file 12 — EV Figures Source Data [file 44319_2024_85_MOESM12_ESM.zip › Figure EV1/E/Western_GammaH2A.X.tif]

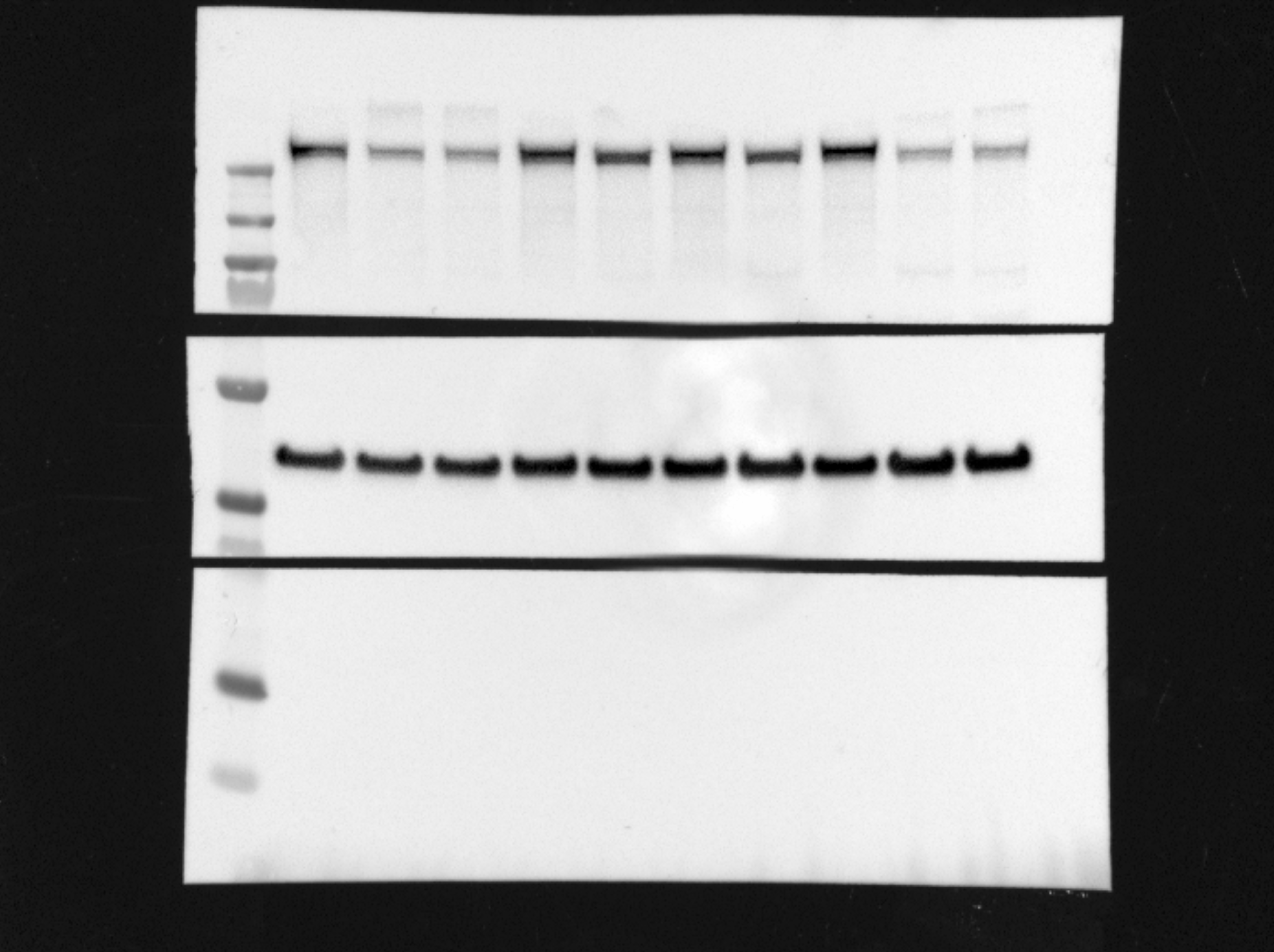

Supplement: Supplementary file 12 — EV Figures Source Data [file 44319_2024_85_MOESM12_ESM.zip › Figure EV1/E/Western_POLR2A_GAPDH.tif]

Figure S1H

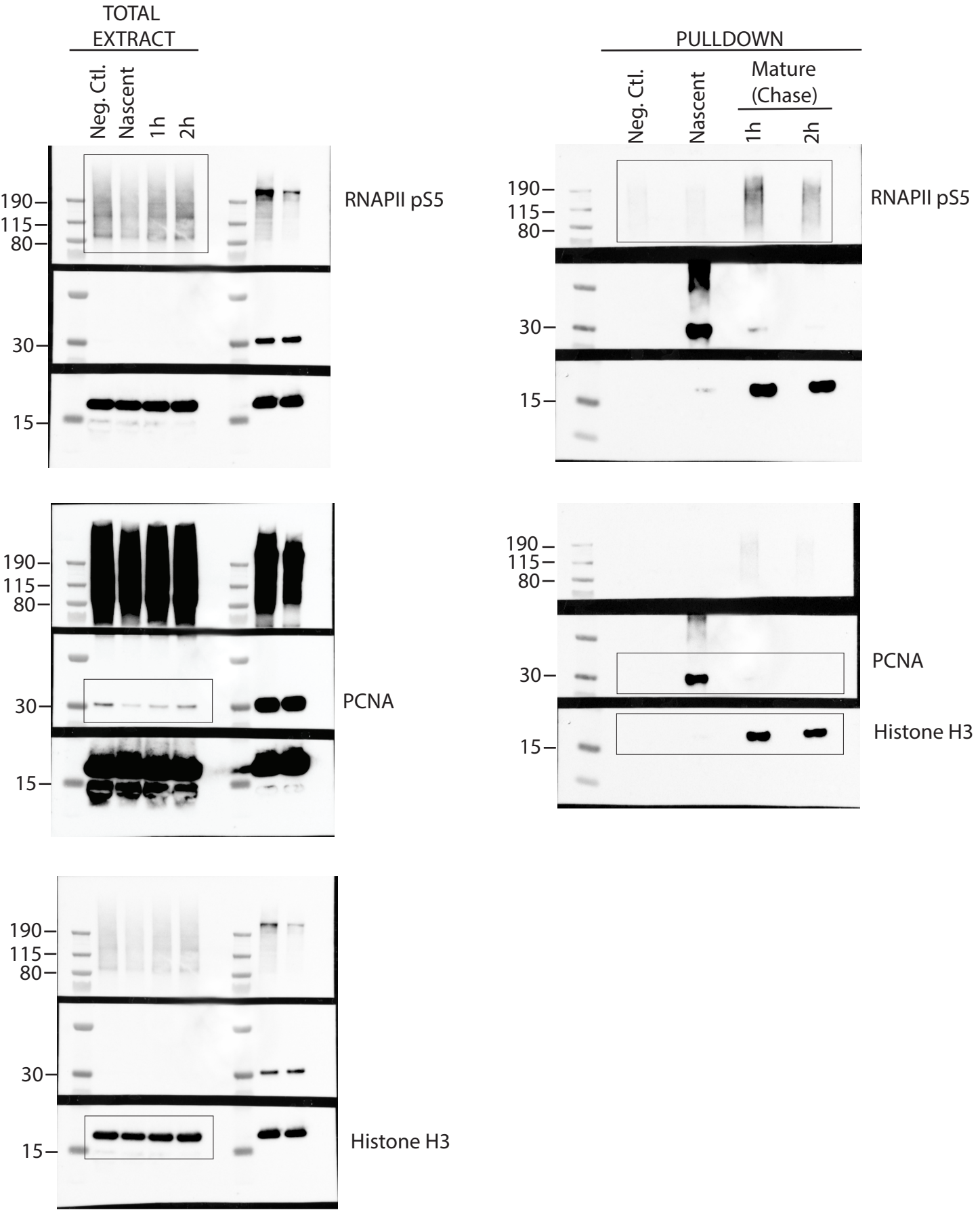

Supplement: Supplementary file 12 — EV Figures Source Data [file 44319_2024_85_MOESM12_ESM.zip › Figure EV1/H/README_WB_S1H.pdf]

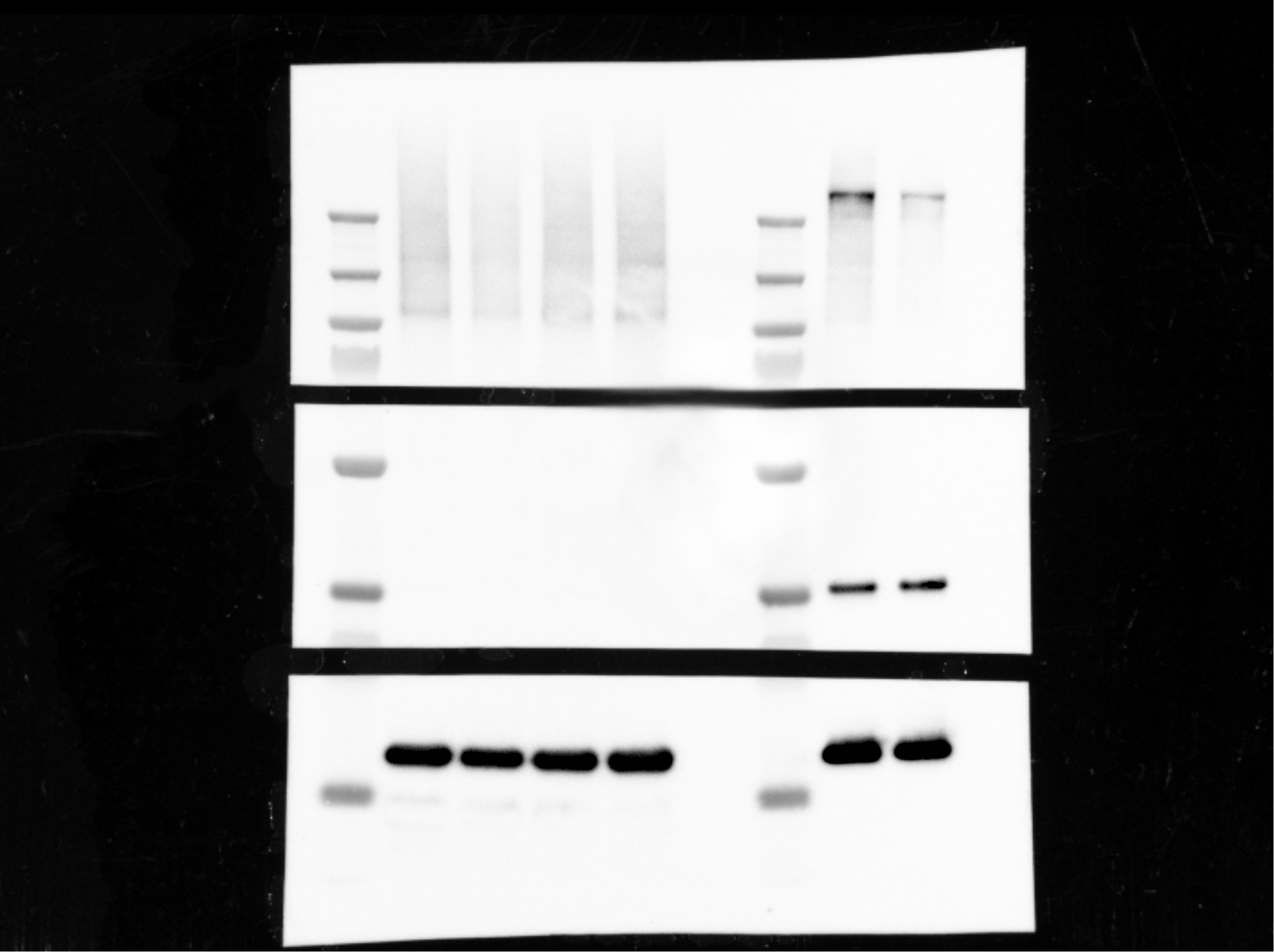

Supplement: Supplementary file 12 — EV Figures Source Data [file 44319_2024_85_MOESM12_ESM.zip › Figure EV1/H/WB_Histone H3_total extract.tif]

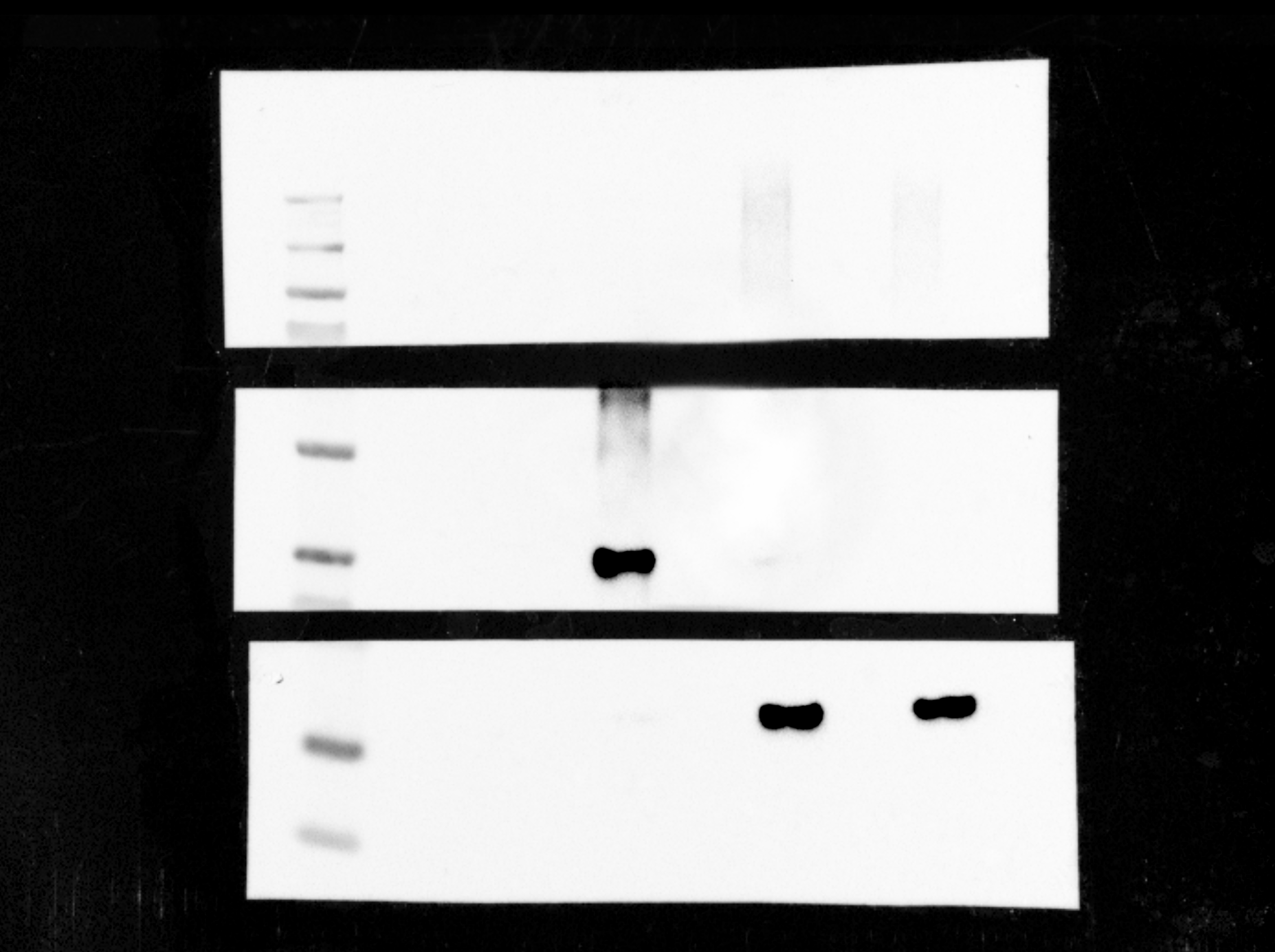

Supplement: Supplementary file 12 — EV Figures Source Data [file 44319_2024_85_MOESM12_ESM.zip › Figure EV1/H/WB_PCNA_Histone H3_iPOND.tif]

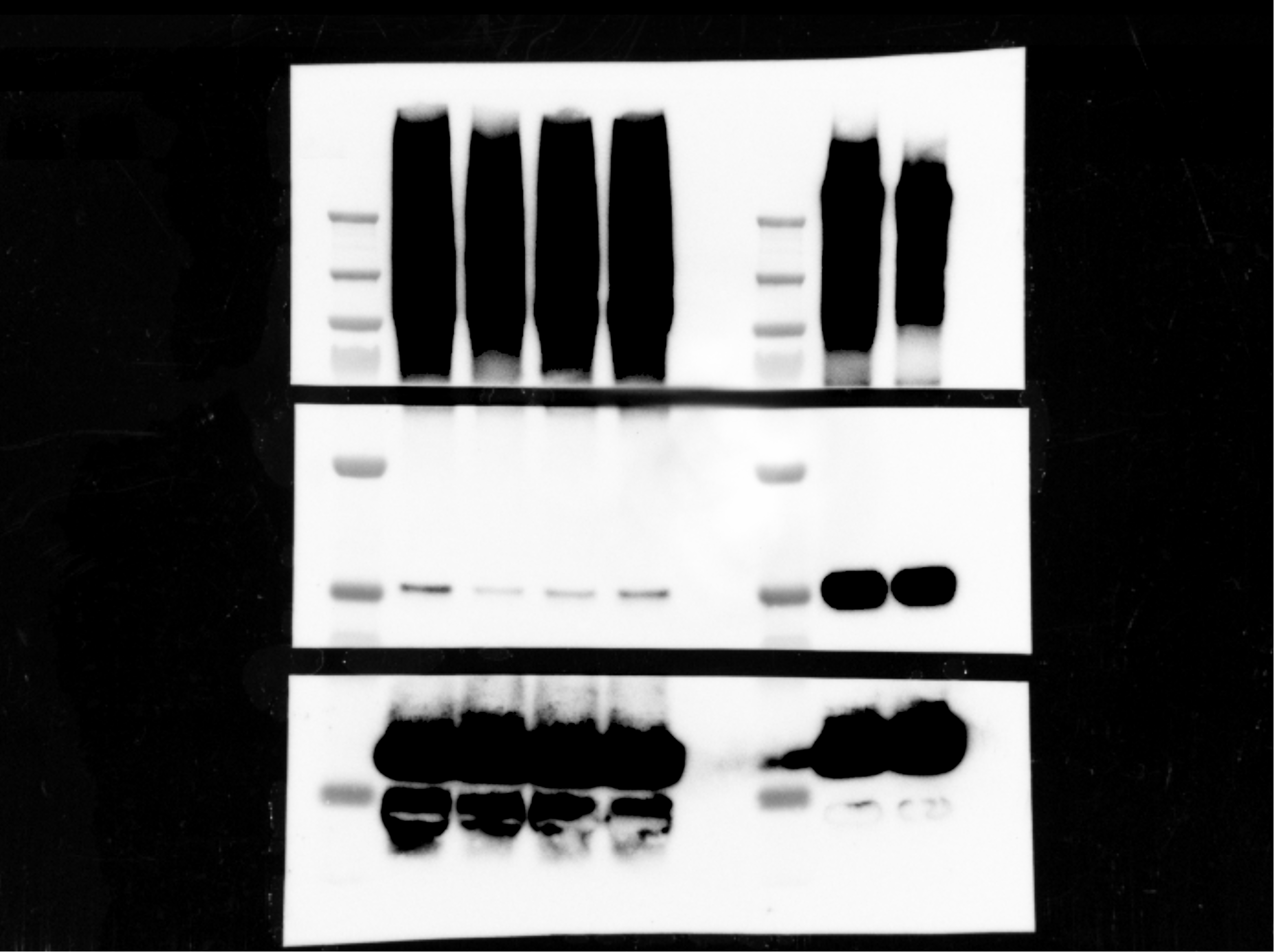

Supplement: Supplementary file 12 — EV Figures Source Data [file 44319_2024_85_MOESM12_ESM.zip › Figure EV1/H/WB_PCNA_total extract.tif]

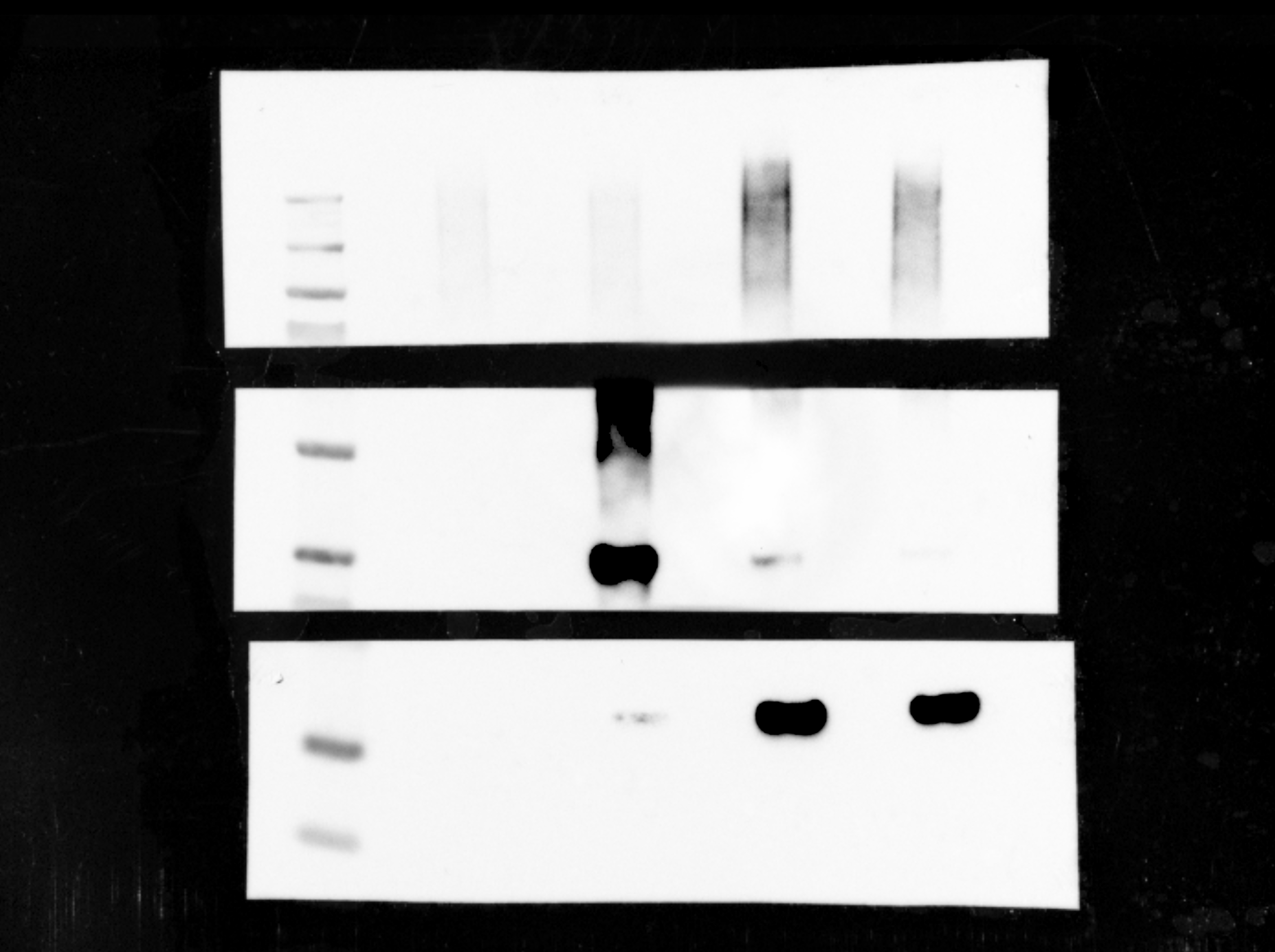

Supplement: Supplementary file 12 — EV Figures Source Data [file 44319_2024_85_MOESM12_ESM.zip › Figure EV1/H/WB_POLR2A-pS5_iPOND.tif]

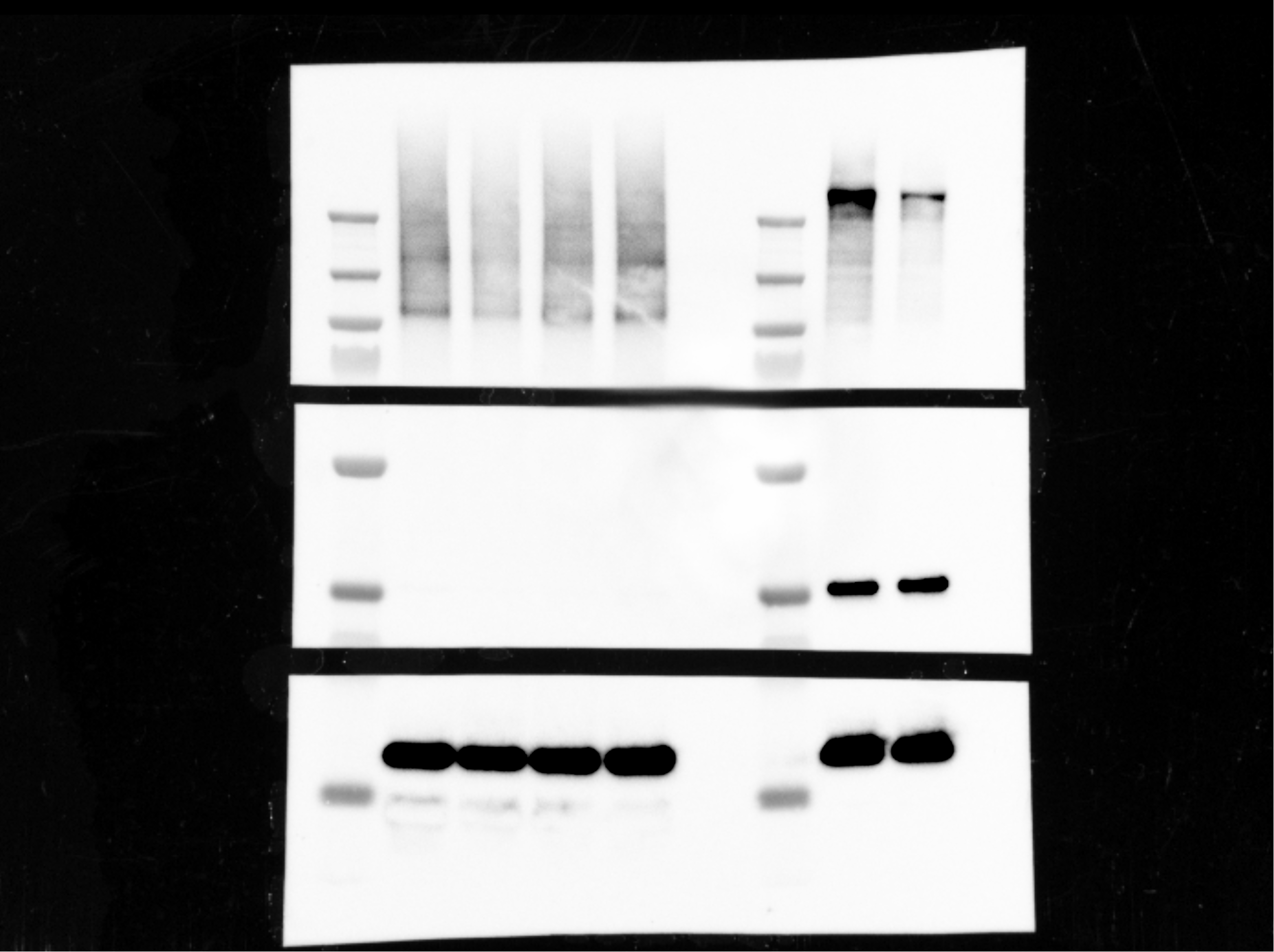

Supplement: Supplementary file 12 — EV Figures Source Data [file 44319_2024_85_MOESM12_ESM.zip › Figure EV1/H/WB_POLR2A-pS5_total extract.tif]

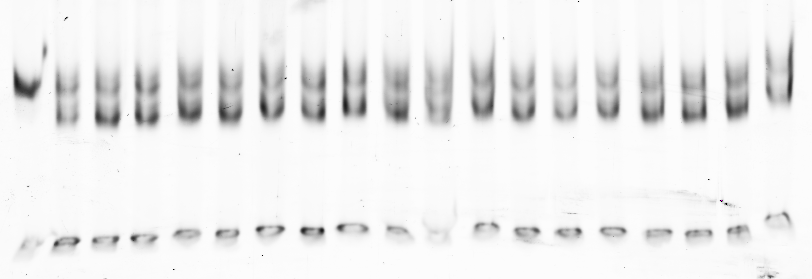

Supplement: Supplementary file 12 — EV Figures Source Data [file 44319_2024_85_MOESM12_ESM.zip › Figure EV6/B/SMARCA4 remodeling gel.PNG]
