# Supplementary material for: RNA polymerase II promotes the organization of chromatin following DNA replication
Source: EMBO Rep. 2024 Feb 12;25(3):1387–414. doi: 10.1038/s44319-024-00085-x (PMC10933433; doi:10.1038/s44319-024-00085-x)
Supplement: Supplementary file 14 — Expanded View Figures [file 44319_2024_85_MOESM14_ESM.pdf]

Expanded View Figures

Figure EV1. Proteomic profiling of chromatin behind replisomes upon transcription inhibition. Related to Fig. 1.

(**A**) QIBC-based analysis of RNAPII level in DMSO, TPL and DRB treated cells. Graphs show the mean intensity per nuclei, > 539 nuclei were analysed per sample. Red line: median. Unpaired Mann–Whitney *t* test; ****P value < 0.0001. $N = 3$ biological replicates, one representative experiment is shown. (**B**) EdU pulse chase quantification by flow cytometry. Cells were pulsed for 11 min with EdU (20 μM) (Nascent sample) and chased 2 h by 20 μM of thymidine (Mature sample). Left, the gating for EdU-positive and negative cells is shown. Right, quantification of cell density (expressed as %) and EdU intensities (a.u.) for 1 N and 2 N populations. (**C**) Flow cytometry plots of asynchronous cells treated with DMSO, TPL and DRB. Cells were EdU pulsed and chased as described in (**B**). Nas, Nascent sample, red; 2 h, Mature sample, black. The percentage of overlapping area between Nas and 2 h is indicated. (**D**) Cells were treated for 2 h with DMSO, TPL and DRB, followed by a 20 min EdU pulse and analysed by microscopy. Left: Representative images of Early/Mid and Late S-phase patterns. Early and Mid-S-phase cells have a pattern of replication foci distributed throughout the nucleus. Late S-phase cells would have a small number of large foci within the nucleus. Right: Distribution of cells in G1 and G2 phase based on EdU and DAPI intensities. Distribution of cells in Early/Mid (top section) and late S phase (bottom section) based on the distinct EdU patterns shown on the left. The mean and standard deviation (S.D.) of >1230 nuclei are shown. $N = 2$ biological replicates. One representative experiment is shown. (**E**) Western plot showing the γH2AX level in cells treated with DMSO, TPL or DRB. Cells were treated with inhibitors for 3 h according to the concentrations indicated. Total cell extracts were collected and GAPDH used as a loading control. (**F**) Principal-component analysis of the four biological replicates based on the proteins identified. (**G**) Volcano plot generated using the iPOND-TMT time course data showing protein fold changes based on the full model for the 2 hr mature time point. $N = 4$ biological replicates, Limma t test, significantly changing proteins with a magnitude fold change ≥0.5 (*x* axis) and an FDR ≤ 0.05 (*y* axis) are highlighted. Replisome, red; Canonical Histones, blue; Histone variants, light blue. The dashed horizontal line shows the *P* value cut-off, and the two vertical dashed lines indicate proteins enriched or depleted from 2 hr mature chromatin. (**H**) Western blot analysis of iPOND samples. The western blot was probed with antibodies against RNAPII-pS5, PCNA, and Histone H3 (indicated on the right). Sample labelling shown on top. (**I**) Heatmap of RNA Polymerase I, II, and II. The log2-fold change of batch-corrected abundance with respect to the protein mean is shown ($n = 4$ biological replicates). Each column represents a time point (N: Nascent, 1 h, and 2 h) and each row corresponds to the protein indicated on the left. Colour scale is indicated. (**J**) Left: Scheme of the PLA analysis between EdU and protein of interest by QIBC. TIG-3 cells are EdU-labelled, and the PLA signal between EdU and the protein of interest on nascent chromatin analysed (see details in 'Methods'). Right: Single-cell PLA signal of EdU-RNAPII interaction shown for nascent chromatin in DMSO, TPL and DRB treated cells. Cells with a similar EdU signal were chosen and the PLA signal was calculated as the SUM of the total intensity of PLA foci per nucleus. >78 nuclei were analysed per sample. Red line, median; Unpaired Mann–Whitney *t* test; ****P value < 0.0001; n.s., non-significant. $N = 2$ biological replicates, one representative experiment is shown. Source data are available online for this figure.

 © The Author(s)

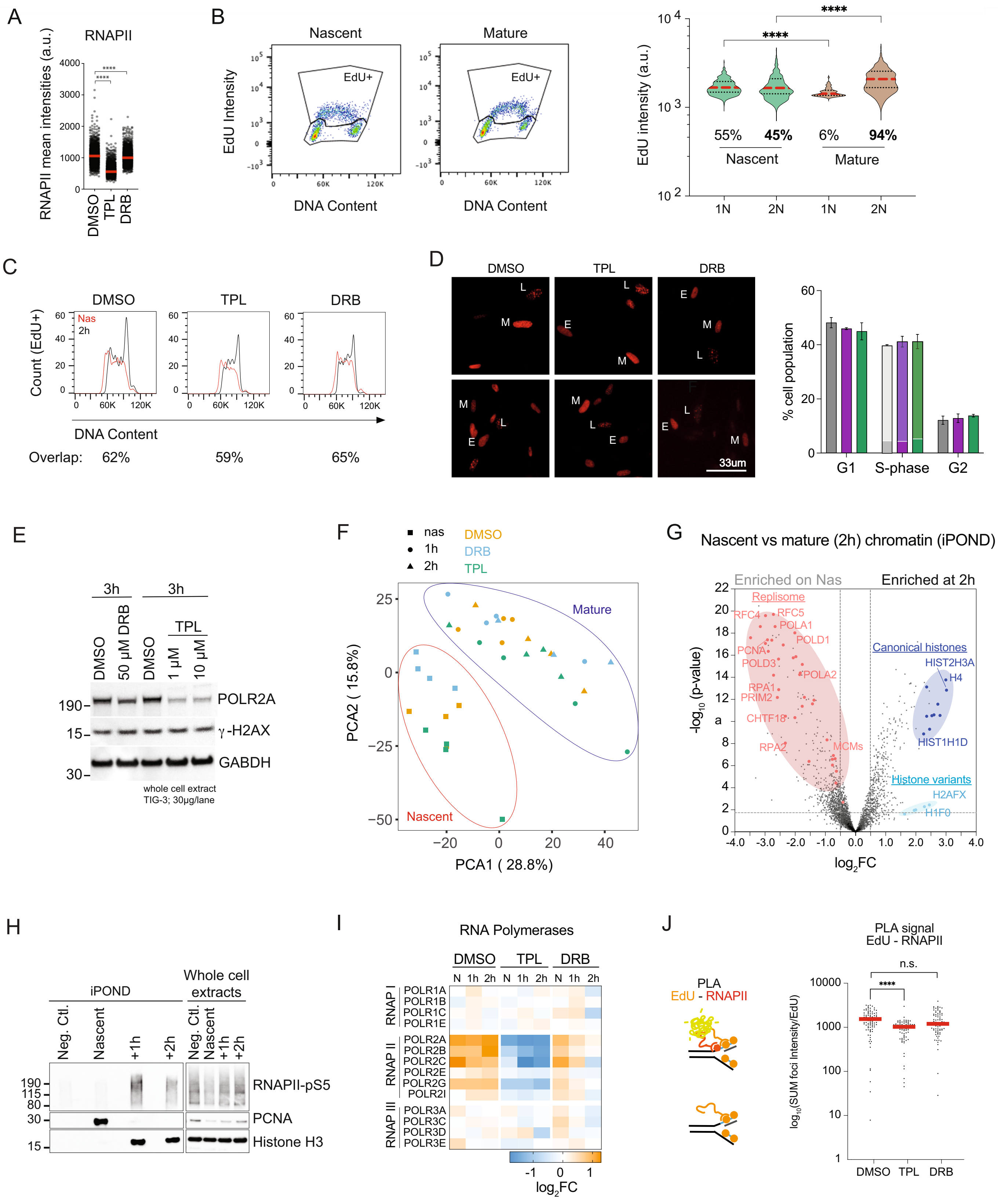

A
RNAPII
RNAPII mean intensities (a.u.)
DMSO
TPL
DRB
B
Nascent
Mature
EdU Intensity
EdU+
DNA Content
EdU intensity (a.u.)
55%
45%
6%
94%
Nascent
Mature
1N
2N
C
DMSO
TPL
DRB
Count (EdU+)
Nas
2h
DNA Content
Overlap: 62% 59% 65%
D
DMSO
TPL
DRB
33um
% cell population
G1
S-phase
G2
E
3h
DMSO
50 µM DRB
TPL
1 µM
10 µM
POLR2A
γ-H2AX
GABDH
whole cell extract
TIG-3; 30µg/lane
F
nas
1h
2h
DMSO
DRB
TPL
Mature
Nascent
PCA2 (15.8%)
PCA1 (28.8%)
G
Nascent vs mature (2h) chromatin (iPOND)
Enriched on Nas
Enriched at 2h
Replisome
Canonical histones
Histone variants
-log10 (p-value)
log2FC
H
iPOND
Whole cell extracts
Neg. Ctl.
Nascent
+1h
+2h
RNAPII-pS5
PCNA
Histone H3
I
RNA Polymerases
DMSO
TPL
DRB
N 1h 2h
RNAP I
RNAP II
RNAP III
log2FC
J
PLA
EdU - RNAPII
PLA signal
EdU - RNAPII
n.s.
log10(SUM foci Intensity/EdU)
DMSO
TPL
DRB

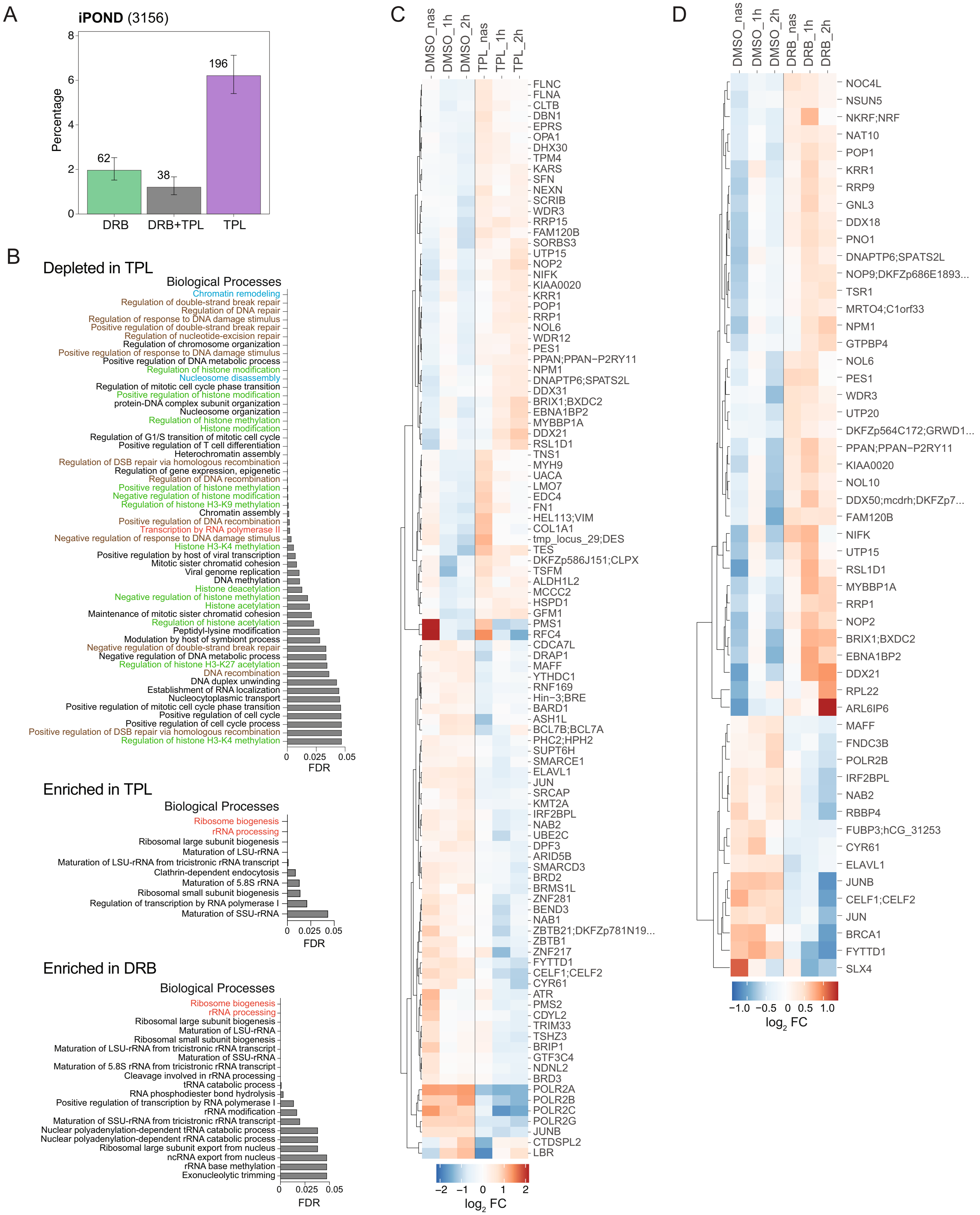
A
iPOND (3156)
Percentage
8
6
4
2
0
62
38
196
DRB
DRB+TPL
TPL
B
Depleted in TPL
Biological Processes
Chromatin remodeling
Regulation of double-strand break repair
Regulation of DNA repair
Regulation of response to DNA damage stimulus
Positive regulation of double-strand break repair
Regulation of nucleotide-excision repair
Regulation of chromosome organization
Positive regulation of response to DNA damage stimulus
Positive regulation of DNA metabolic process
Regulation of histone modification
Nucleosome disassembly
Regulation of mitotic cell cycle phase transition
Positive regulation of histone modification
protein-DNA complex subunit organization
Nucleosome organization
Regulation of histone methylation
Histone modification
Regulation of G1/S transition of mitotic cell cycle
Positive regulation of T cell differentiation
Heterochromatin assembly
Regulation of DSB repair via homologous recombination
Regulation of gene expression, epigenetic
Regulation of DNA recombination
Positive regulation of histone methylation
Negative regulation of histone modification
Regulation of histone H3-K9 methylation
Chromatin assembly
Positive regulation of DNA recombination
Transcription by RNA polymerase II
Negative regulation of response to DNA damage stimulus
Histone H3-K4 methylation
Positive regulation by host of viral transcription
Mitotic sister chromatid cohesion
Viral genome replication
DNA methylation
Histone deacetylation
Negative regulation of histone methylation
Histone acetylation
Maintenance of mitotic sister chromatid cohesion
Regulation of histone acetylation
Peptidyl-lysine modification
Modulation by host of symbiont process
Negative regulation of double-strand break repair
Negative regulation of DNA metabolic process
Regulation of histone H3-K27 acetylation
DNA recombination
DNA duplex unwinding
Establishment of RNA localization
Nucleocytoplasmic transport
Positive regulation of mitotic cell cycle phase transition
Positive regulation of cell cycle
Positive regulation of cell cycle process
Positive regulation of DSB repair via homologous recombination
Regulation of histone H3-K4 methylation
0 0.025 0.05
FDR
Enriched in TPL
Biological Processes
Ribosome biogenesis
rRNA processing
Ribosomal large subunit biogenesis
Maturation of LSU-rRNA
Maturation of LSU-rRNA from tricistronic rRNA transcript
Clathrin-dependent endocytosis
Maturation of 5.8S rRNA
Ribosomal small subunit biogenesis
Regulation of transcription by RNA polymerase I
Maturation of SSU-rRNA
0 0.025 0.05
FDR
Enriched in DRB
Biological Processes
Ribosome biogenesis
rRNA processing
Ribosomal large subunit biogenesis
Maturation of LSU-rRNA
Ribosomal small subunit biogenesis
Maturation of LSU-rRNA from tricistronic rRNA transcript
Maturation of SSU-rRNA
Maturation of 5.8S rRNA from tricistronic rRNA transcript
Cleavage involved in rRNA processing
tRNA catabolic process
RNA phosphodiester bond hydrolysis
Positive regulation of transcription by RNA polymerase I
rRNA modification
Maturation of SSU-rRNA from tricistronic rRNA transcript
Nuclear polyadenylation-dependent tRNA catabolic process
Nuclear polyadenylation-dependent rRNA catabolic process
Ribosomal large subunit export from nucleus
ncRNA export from nucleus
rRNA base methylation
Exonucleolytic trimming
0 0.025 0.05
FDR
C
DMSO_nas DMSO_1h DMSO_2h TPL_nas TPL_1h TPL_2h
FLNC
FLNA
CLTB
DBN1
EPRS
OPA1
DHX30
TPM4
KARS
SFN
NEXN
SCRIB
WDR3
RRP15
FAM120B
SORBS3
UTP15
NOP2
NIFK
KIAA0020
KRR1
POP1
RRP1
NOL6
WDR12
PES1
PPAN;PPAN−P2RY11
NPM1
DNAPTP6;SPATS2L
DDX31
BRIX1;BXDC2
EBNA1BP2
MYBBP1A
DDX21
RSL1D1
TNS1
MYH9
UACA
LMO7
EDC4
FN1
HEL113;VIM
COL1A1
tmp_locus_29;DES
TES
DKFZp586J151;CLPX
TSFM
ALDH1L2
MCCC2
HSPD1
GFM1
PMS1
RFC4
CDCA7L
DRAP1
MAFF
YTHDC1
RNF169
Hin−3;BRE
BARD1
ASH1L
BCL7B;BCL7A
PHC2;HPH2
SUPT6H
SMARCE1
ELAVL1
JUN
SRCAP
KMT2A
IRF2BPL
NAB2
UBE2C
DPF3
ARID5B
SMARCD3
BRD2
BRMS1L
ZNF281
BEND3
NAB1
ZBTB21;DKFZp781N19...
ZBTB1
ZNF217
FYTTD1
CELF1;CELF2
CYR61
ATR
PMS2
CDYL2
TRIM33
TSHZ3
BRIP1
GTF3C4
NDNL2
BRD3
POLR2A
POLR2B
POLR2C
POLR2G
JUNB
CTDSPL2
LBR
−2 −1 0 1 2
$\log_2$ FC
D
DMSO_nas DMSO_1h DMSO_2h DRB_nas DRB_1h DRB_2h
NOC4L
NSUN5
NKRF;NRF
NAT10
POP1
KRR1
RRP9
GNL3
DDX18
PNO1
DNAPTP6;SPATS2L
NOP9;DKFZp686E1893...
TSR1
MRTO4;C1orf33
NPM1
GTPBP4
NOL6
PES1
WDR3
UTP20
DKFZp564C172;GRWD1...
PPAN;PPAN−P2RY11
KIAA0020
NOL10
DDX50;mcdrh;DKFZp7...
FAM120B
NIFK
UTP15
RSL1D1
MYBBP1A
RRP1
NOP2
BRIX1;BXDC2
EBNA1BP2
DDX21
RPL22
ARL6IP6
MAFF
FNDC3B
POLR2B
IRF2BPL
NAB2
RBBP4
FUBP3;hCG_31253
CYR61
ELAVL1
JUNB
CELF1;CELF2
JUN
BRCA1
FYTTD1
SLX4
−1.0 −0.5 0 0.5 1.0
$\log_2$ FC

 © The Author(s)

Figure EV2. Proteomic profiling of chromatin behind replisomes upon transcription inhibition. Related to Fig. 1.

(**A**) Histogram showing the number of factors that had a significant fold change based on the full model for TPL and DRB treatment shown in Fig. 1F,G. TPL + DRB: common factors between TPL and DRB treatment. $N = 4$ biological replicates. 95% confidence interval of a proportion is shown. (**B**) GO term analysis using STRING (Szklarczyk et al, 2015) with the significantly changing proteins based on the full model for TPL and DRB treatment shown in Fig. 1F,G. GO terms were filtered for a background gene count <400, chromatin related biological processes and an FDR below 0.05. The proteins were categorised into three groups: Depleted in TPL, enriched in TPL, and enriched in DRB. The GO terms have been colour coded based on four categories: Chromatin remodelling, blue; DNA repair, brown; Histone modification, green; Transcription, red. (**C**, **D**) The rows in the heatmaps were clustered using complete-linkage hierarchical clustering with Euclidean distance metric (see material and method for details). Source data are available online for this figure.

© The Author(s)

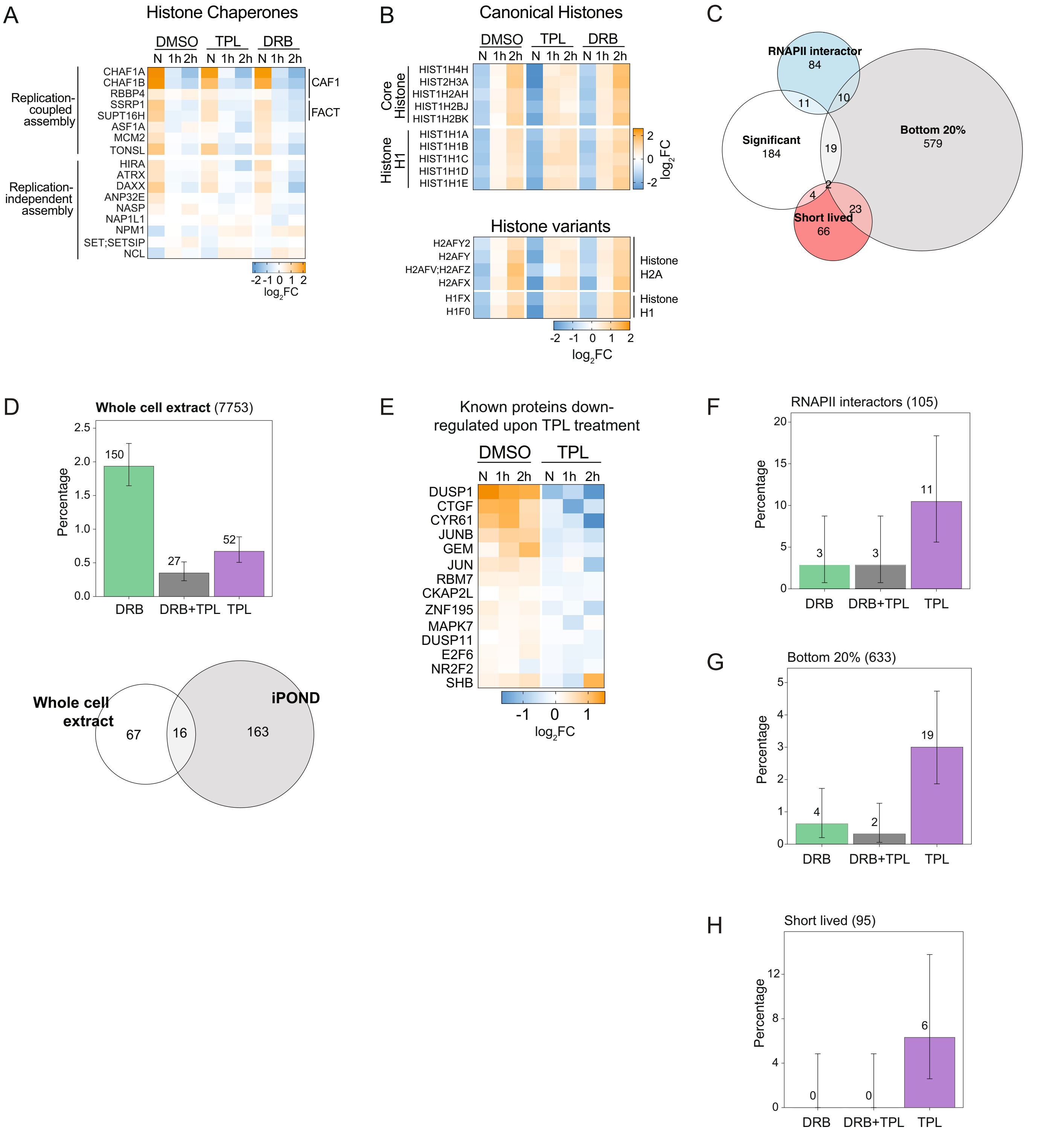
A
Histone Chaperones
DMSO TPL DRB
N 1h 2h N 1h 2h N 1h 2h
Replication-coupled assembly
CHAF1A
CHAF1B
RBBP4
SSRP1
SUPT16H
ASF1A
MCM2
TONSL
CAF1
FACT
Replication-independent assembly
HIRA
ATRX
DAXX
ANP32E
NASP
NAP1L1
NPM1
SET;SETSIP
NCL
-2 -1 0 1 2
log2FC
B
Canonical Histones
DMSO TPL DRB
N 1h 2h N 1h 2h N 1h 2h
Core Histone
HIST1H4H
HIST2H3A
HIST1H2AH
HIST1H2BJ
HIST1H2BK
Histone H1
HIST1H1A
HIST1H1B
HIST1H1C
HIST1H1D
HIST1H1E
2
0
-2
log2FC
Histone variants
H2AFY2
H2AFY
H2AFV;H2AFZ
H2AFX
H1FX
H1F0
Histone H2A
Histone H1
-2 -1 0 1 2
log2FC
C
RNAPII interactor
84
10
11
Significant
184
19
Bottom 20%
579
4
2
23
Short lived
66
D
Whole cell extract (7753)
Percentage
2.5
2.0
1.5
1.0
0.5
0.0
150
27
52
DRB
DRB+TPL
TPL
Whole cell extract
67
16
iPOND
163
E
Known proteins down-regulated upon TPL treatment
DMSO TPL
N 1h 2h N 1h 2h
DUSP1
CTGF
CYR61
JUNB
GEM
JUN
RBM7
CKAP2L
ZNF195
MAPK7
DUSP11
E2F6
NR2F2
SHB
-1 0 1
log2FC
F
RNAPII interactors (105)
Percentage
20
15
10
5
0
3
3
11
DRB
DRB+TPL
TPL
G
Bottom 20% (633)
Percentage
5
4
3
2
1
0
4
2
19
DRB
DRB+TPL
TPL
H
Short lived (95)
Percentage
12
8
4
0
0
0
6
DRB
DRB+TPL
TPL

 © The Author(s)

Figure EV3. Proteomic profiling of chromatin behind replisomes upon transcription inhibition. Related to Fig. 1.

(**A**, **B**) Heatmaps of Histone Chaperones (**A**) and Canonical Histones/Histone variants (**B**). The log2-fold change of batch-corrected abundance with respect to the protein mean is shown ($n = 4$ biological replicates). Each column represents a time point (N: Nascent, 1 h, and 2 h) and each row corresponds to the protein indicated on the left. Colour scale is indicated. (**C**) Venn diagram between proteins with a significant fold change based on the full model for TPL and DRB treatment (white, Fig. 1F/G), identified RNAPII interactor (blue), identified short-lived proteins (red), and the 20% lowest abundant proteins identified (light purple). The lists of RNAPII interactor and short-lived proteins were generated based on (Ebmeier et al, 2017) and (Li et al, 2021), respectively. (**D**) Top: Same as in Fig. EV2A for significantly changing proteins in the whole-cell extract upon TPL or DRB treatment (shown in Fig. 1H,I). 95% confidence interval of a proportion is shown. Bottom: Venn diagram between significantly changing proteins (FDR < 0.05, full model for TPL and DRB treatment) from the whole-cell extract and the iPOND-TMT time course experiments. $N = 4$ biological replicates. (**E**) Same as in (**A**, **B**) for reported proteins by (Vispe et al, 2009) downregulated upon TPL treatment. $N = 4$ biological replicates. (**F–H**) Same as in Fig. EV2A for identified RNAPII interactor (**F**), the 20% lowest abundant proteins (**G**) and the identified short-lived proteins (**H**). $N = 4$ biological replicates. 95% confidence interval of a proportion is shown.

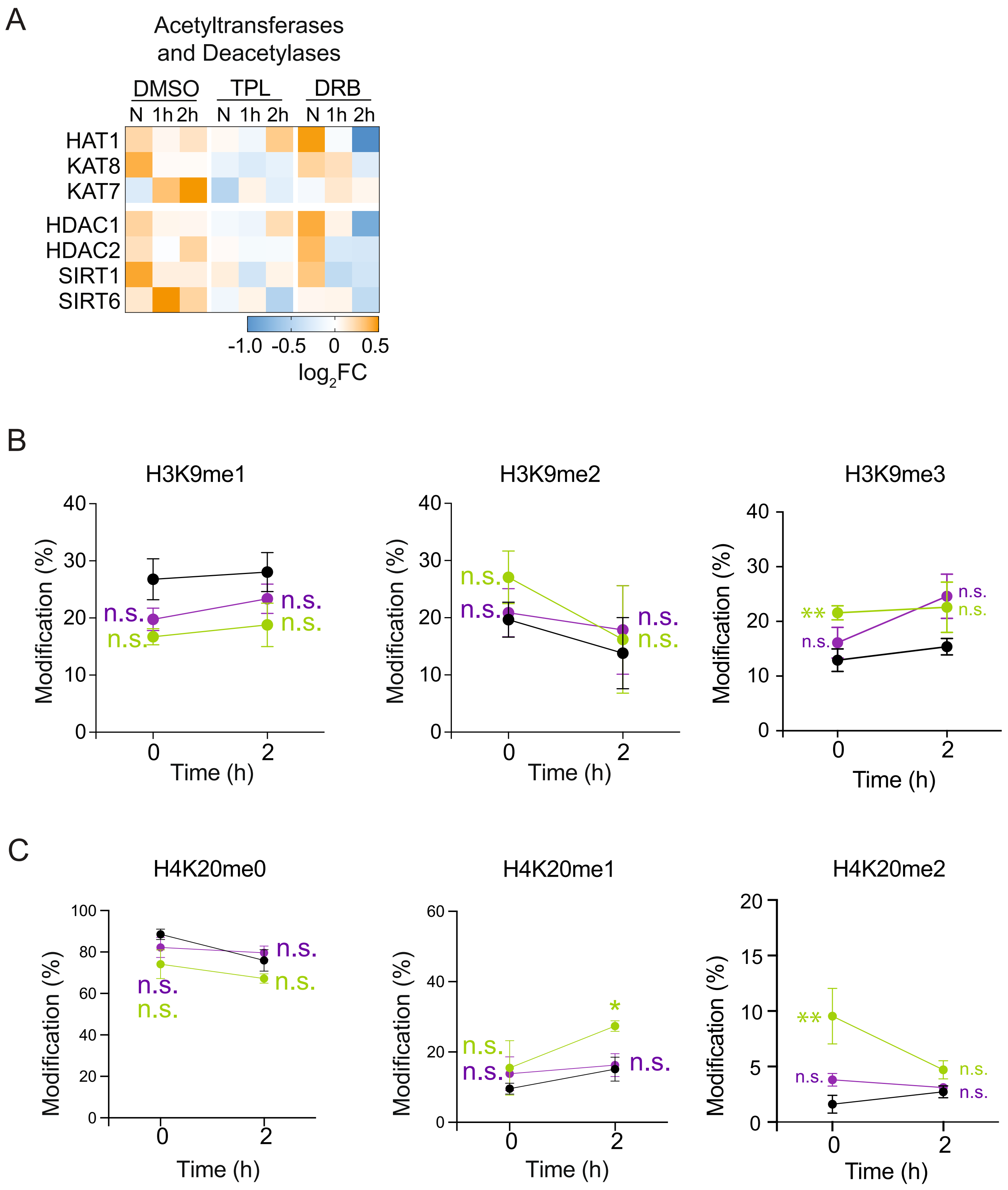
A
Acetyltransferases and Deacetylases
DMSO TPL DRB
N 1h 2h N 1h 2h N 1h 2h
HAT1
KAT8
KAT7
HDAC1
HDAC2
SIRT1
SIRT6
-1.0 -0.5 0 0.5
log2FC
B
H3K9me1
H3K9me2
H3K9me3
Modification (%)
Time (h)
n.s.
**
C
H4K20me0
H4K20me1
H4K20me2
*

 © The Author(s)

Figure EV4. Transcription promotes H3.3K36me2 re-establishment on replicated chromatin. Related to Fig. 2.

(**A**) Heatmap of Histone Acetyltransferases and Deacetylates ($n = 4$ biological replicates). The log2-fold change of batch-corrected abundance with respect to the protein mean is shown ($n = 4$ biological replicates). Each column represents a time point (N: Nascent, 1 h, and 2 h) and each row corresponds to the protein indicated on the left. Colour scale is indicated. (**B**) Proportion of H3K9me1, H3K9me2, and H3K9me3 (left to right) on newly replicated chromatin for each time point in DMSO (black), DRB (green) and TPL (purple) treated cells. Standard error of the mean is shown. $N = 3$ biological replicates. Paired t test, **P value < 0.01; n.s., non-significant. (**C**) Same as in (**B**) for H4K20me0, H4K20me1, and H4K20me2 (left to right).

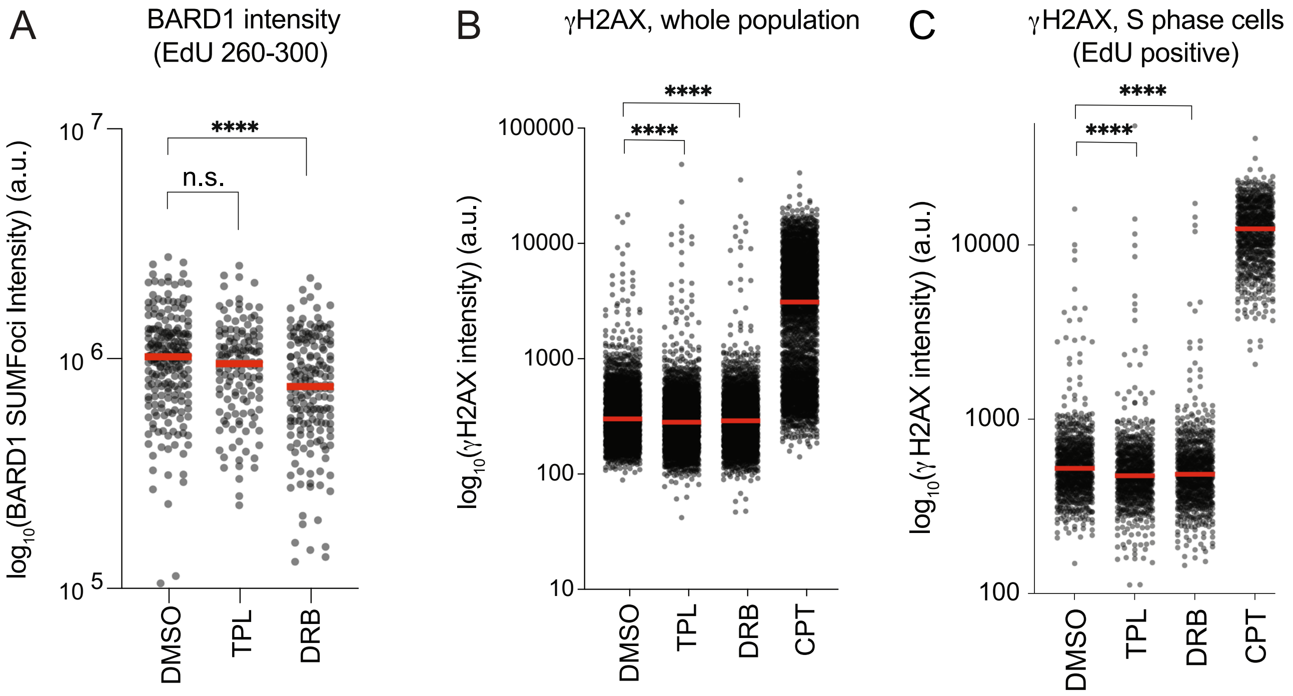

Figure EV5. Blocking transcription reduced the recruitment of DNA repair proteins on replicated chromatin. Related to Fig. 3.

(**A**) Single-cell PLA signal of EdU-BARD1 interaction shown for nascent chromatin in DMSO, TPL and DRB treated cells. Cells with a similar EdU signal were chosen and the PLA signal was calculated as the SUM of the total intensity of PLA foci per nucleus. >138 nuclei were analysed per sample. Red line, median; Unpaired Mann–Whitney *t* test; ****P value < 0.0001; n.s., non-significant. $N = 2$ biological replicates, one representative experiment is shown. (**B**, **C**) Quantification of γH2AX signal in the whole single-cell population (**B**) and EdU-positive cells only (**C**). Quantification of γH2AX fluorescence signals in individual cells was measured by QIBC. Cells were treated with TPL and DRB according to conditions used in the study and labelled with EdU for the last 20 min. Camptothecin treatment is used as a positive control for DNA damage. >600 nuclei were analysed per condition. Red line, median; Unpaired Mann–Whitney *t* test; ****P value < 0.0001. $N = 2$ biological replicates, one representative experiment is shown. Source data are available online for this figure.

© The Author(s)

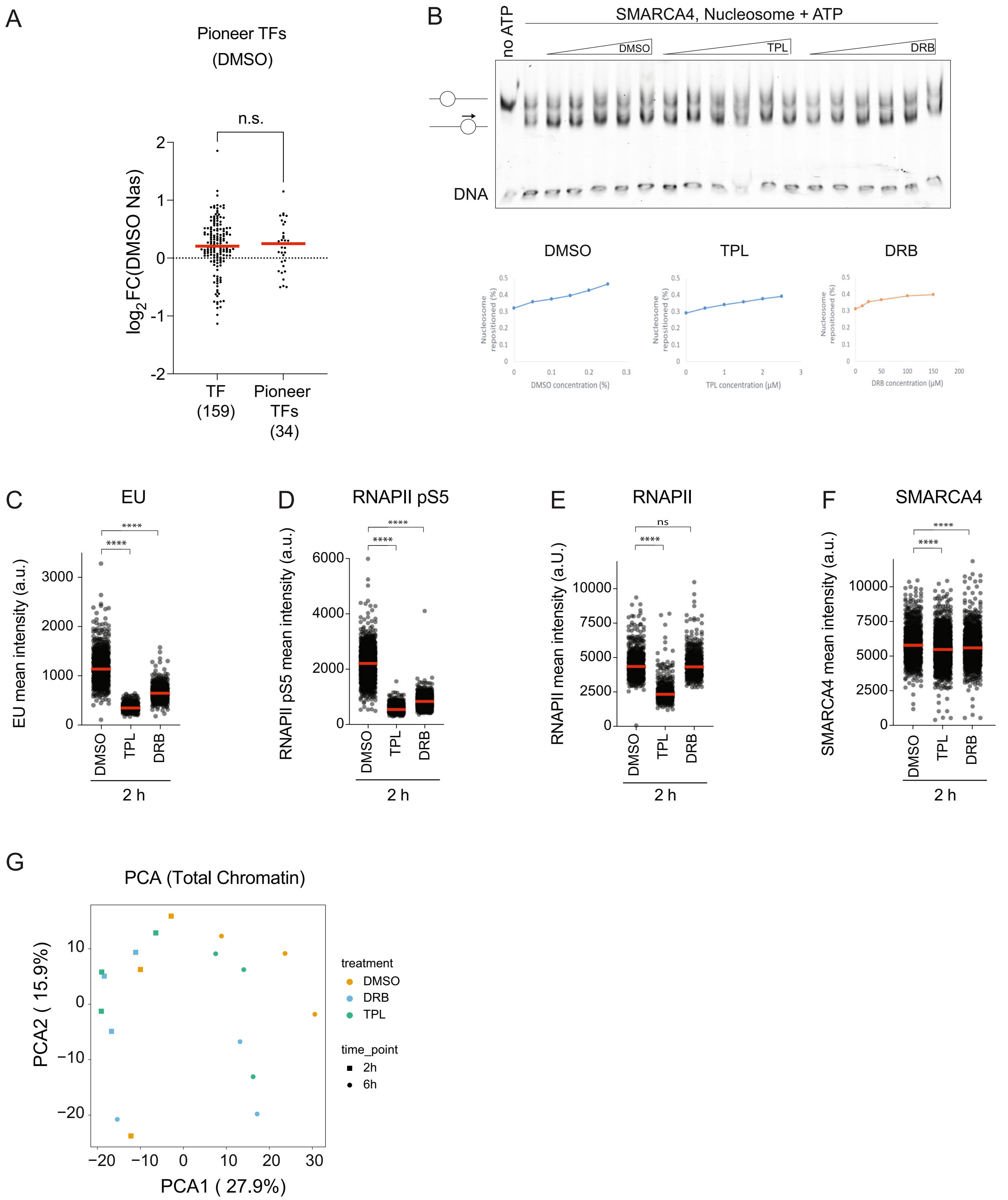
A
Pioneer TFs
(DMSO)
n.s.
log2FC(DMSO Nas)
TF
(159)
Pioneer
TFs
(34)
B
no ATP
SMARCA4, Nucleosome + ATP
DMSO
TPL
DRB
DNA
DMSO
TPL
DRB
Nucleosome repositioned (%)
DMSO concentration (%)
TPL concentration (μM)
DRB concentration (μM)
C
EU
EU mean intensity (a.u.)
D
RNAPII pS5
RNAPII pS5 mean intensity (a.u.)
E
RNAPII
RNAPII mean intensity (a.U.)
F
SMARCA4
SMARCA4 mean intensity (a.u.)
DMSO
TPL
DRB
2 h
****
ns
G
PCA (Total Chromatin)
PCA2 (15.9%)
PCA1 (27.9%)
treatment
DMSO
DRB
TPL
time_point
2h
6h

Figure EV6. Analysis of TF and remodellers on replicated chromatin and of steady state chromatin. Related to Figs. 4, 5 and 6.

(**A**) Log_2 fold change of batch-corrected abundance with respect to the protein mean for the nascent time point is shown ($n = 4$ biological replicates). TFs are divided into two groups, pioneer factors and non-pioneer (Sherwood et al, 2014). Red line, median; Unpaired Mann–Whitney *t* test; n.s., non-significant. (**B**) Nucleosome sliding in vitro assay (left) and its quantification (right). (**C–F**) QIBC analysis of chromatin-bound intensities of EU, RNAPII-pS5, RNAPII and SMARCA4 in DMSO, TPL or DRB treated cells. Graphs show the mean intensity per nuclei, >291 nuclei were analysed per sample. Red line, median; Unpaired Mann–Whitney *t* test; ****P value < 0.0001; n.s., non-significant. $N = 3$ biological replicates, one representative experiment is shown. (**G**) Principal-component analysis using the identified proteins from the iPOND-TMT chase experiments. Source data are available online for this figure.

 © The Author(s)
